# Childbearing and mortality among women with personality disorders: nationwide registered-based cohort study

**DOI:** 10.1192/bjo.2020.77

**Published:** 2020-08-25

**Authors:** Efthymios Kouppis, Charlotte Björkenstam, Bengt Gerdin, Lisa Ekselius, Emma Björkenstam

**Affiliations:** Department of Neuroscience, Uppsala University, Sweden; Department of Neuroscience, Uppsala University, Sweden; Department of Surgical Sciences, Uppsala University, Sweden; Department of Neuroscience, Uppsala University, Sweden; Department of Neuroscience, Uppsala University, Sweden; and Division of Insurance Medicine, Department of Clinical Neuroscience, Karolinska Institutet, Sweden

**Keywords:** Personality disorder, childbearing, women, national registers, mortality

## Abstract

**Background:**

People with a personality disorder have a higher mortality and reduced life expectancy than the general population. Childbearing is thought to have a protective effect on morbidity and mortality. Yet, there are no studies on whether childbearing is related to a lower mortality among women with personality disorder.

**Aims:**

This study examined associations between childbearing and mortality among women with personality disorder. Our hypothesis was that parity would be associated with lower mortality.

**Method:**

This register-based cohort study included 27 412 women treated for personality disorder in in-patient or specialised out-patient care between 1990 and 2015. We used nationwide population-based registers to obtain information on sociodemographics, child delivery, healthcare use and mortality. Mortality risk estimates were calculated as hazard ratios (HRs) with 95% CIs using Cox regression. Adjustments were made for year of birth, educational level, age at diagnosis, comorbidity and severity of personality disorder.

**Results:**

Nulliparous women had a nearly twofold increased mortality risk (adjusted HR = 1.78, 95% CI 1.50–2.12) compared with parous women and over twofold mortality risk (adjusted HR = 2.29, 95% CI 1.72–3.04) compared with those giving birth after their first personality disorder diagnosis. Those giving birth before their first personality disorder diagnosis had a 1.5-fold higher risk of mortality than those giving birth after their first personality disorder diagnosis (adjusted HR = 1.48, 95% CI 1.06–2.07). There was a threefold risk of suicide in nulliparous women compared with those giving birth after their first personality disorder diagnosis (adjusted HR = 2.90, 95% CI 1.97–4.26).

**Conclusions:**

Childbearing history should be an integral part of the clinical evaluation of women with personality disorder.

## Background

Personality disorders represent a global health problem and are estimated to occur in more than 5% of adults worldwide.^1^ It is well established that individuals with one or more personality disorders have higher all-cause mortality and a reduced life expectancy compared with the general population.^2–4^ Moreover, they have an increased risk of death because of homicide, suicide or accidents.^3–5^ Comorbidity with other psychiatric^6,7^ and physical conditions^8^ is common, and contribute to the excess mortality.^3,4,9^ The prevalence of personality disorder is similar in women and men^10^ but more women are treated in healthcare.^3,4^

## Influence of life events on expression of personality disorder

An issue not fully addressed is how life events influence the expression of personality disorder in affected women. This issue also applies to the multitude of reproductive factors that are part of a woman's life. Pregnancy is probably the reproductive factor that has the greatest effect on a woman's life.

Typically, pregnancy has been seen as a time of emotional well-being and is related to lower mortality risk in the general population.^11–13^ One Australian study^11^ clearly shows that all-cause mortality in women decreased with increasing parity. Another study from Japan^12^ showed similar findings, i.e. lower all-cause mortality in parous women. Last, in a meta-analysis from 2016,^13^ participants with no live birth had higher risk of all-cause mortality compared with participants with one or more live births. Childbearing has also been linked to a lower risk of suicide, particularly when children are small,^14^ as well as after adjustment for psychiatric morbidity.^15^ Moreover, childbearing is associated with a 65% lower mortality in patients with anorexia nervosa.^16^ However, the consequence of a pregnancy for a woman is highly contextual. A large proportion of women experience mood or anxiety disorders during pregnancy.^17^ Particularly vulnerable are women with a history of psychiatric illness^18,19^ and those who discontinue psychotropic medication^20,21^ during pregnancy.

Childbearing women with avoidant, dependent and obsessive–compulsive personality disorders have an increased risk of major depression during the postpartum period.^22^ Finally, teenage mothers, independent of socioeconomic background, face an increased risk of early death.^23,24^

Pregnancies in women with borderline personality disorder are suggested to occur earlier than in the background population,^25,26^ to be more unintended^25^ and related to more adverse maternal and fetal outcomes.^26^ The overall consequences of childbearing for mothers with a wider diagnosis of personality disorder are unknown, however.

## Aims

We hypothesised that, even after considering stress load, bearing a child would represent a better prognosis in women diagnosed with personality disorder. Using the Swedish national registers, we enrolled a large nationwide cohort of 27 412 women diagnosed with personality disorder between 1990 and 2015. We aimed to investigate whether giving birth was related to a lower overall and cause-specific mortality in women with a personality disorder. As a secondary aim, we also examined whether women who gave birth before the personality disorder diagnosis had a higher risk of mortality than those who gave birth after being diagnosed with personality disorder.

## Method

### Study population

The study population was defined as all women treated in an in-patient or specialised out-patient unit in the Swedish healthcare system with a diagnosis of personality disorder between and including the years 1990 and 2015, and who were 15–64 years at the time of diagnosis (*n* = 50 607). Patients born before 1971 (*n* = 22 648) were excluded as we had no information about their delivery status nor their personality disorder status. Those born after 1995 (*n* = 431) were also excluded because these women were too young to have been exposed, i.e. to have given birth. Last, those who were diagnosed with personality disorder before 1990 (*n* = 116) were also excluded in order for us to have incident personality disorder ‘cases’. After applying these exclusion criteria, 27 412 women were included in the final cohort.

The participants were identified from the National Patient Register (NPR). This register covers all individuals admitted to psychiatric or general hospital care since 1973 or who had medical appointments in specialised out-patient care since 2001. The NPR has almost complete coverage^27^ and has good validity for personality disorder diagnoses.^28^ Diagnoses in the NPR are coded according to the ICD-10.^29^ The Medical Birth Register was used to obtain information about parity. This register contains data on all deliveries in Sweden since 1973. The Cause of Death Register was used to obtain information about the cause of death. The register includes details of all people who have died in Sweden since 1952. The validity of this register is high (the cause of death is missing in about 1% of the deaths).^30^ The underlying causes of death were coded according to the ICD-10. Finally, information about education level was obtained from the Participation in Education Register.^31^

### Ethics

The study was approved by the Regional Ethics Review Board of Uppsala (dnr: 2013/2028–31/5). Informed consent was waived by the board because the study was strictly register based. The authors assert that all procedures contributing to this work comply with the ethical standards of the relevant national and institutional committees on human experimentation and with the Helsinki Declaration of 1975, as revised in 2008.

### Exposure

Classification of personality disorder and comorbidity were coded according to ICD-9^32^ between 1989 and 1996 and ICD-10 from 1997 onwards. The following ICD-codes were used: ICD-9: 301.0–301.9, and ICD-10: F60.0–F60.9.

Information about birth-giving from 1973 onwards was obtained from the Medical Birth Register. We compared nulliparous with parous women. In addition, we compared those who delivered before versus after the diagnosis of personality disorder.

### Outcome

Additional to examining all-cause mortality, we assessed natural and unnatural death separately. Suicide (ICD-9: E950–E959 and E980–989, ICD-10: X60–X84 and Y10–Y34) was studied as a separate outcome. Inclusion of undetermined intent in the measure of suicide and suicide attempt reduces underreporting and spatial and secular trends in detecting and classifying cases of suicide attempt and suicide when intent was indeterminable.^33^

### Confounders

Education level, used as a proxy for socioeconomic status, was categorised into three groups: compulsory school (≤9 years of education), high school (10–12 years) and college or university (≥13 years). The highest educational level, available for every individual was taken into account in analyses. Given that individuals with personality disorder usually have turbulent lifelines leading to radical changes of many socioeconomic estimates during the lifetime, we considered educational level as more representative than other factors of socioeconomic status. The highest educational level obtained by an individual is a more consistent indicator of the person's overall performance skills and is in bibliography strongly connected with health-related behavioural patterns.^34^

The severity of the underlying condition was considered ‘high’ for those admitted to in-patient care at any time after the first contact, and ‘low’ for those who had only specialised out-patient care. All coexisting somatic diagnoses registered in the NPR after the first personality disorder, except for the ICD-9 codes 650–670 and ICD-10 O-codes representing conditions related to pregnancy, childbirth and puerperium, were reported as a comorbidity, as were any coexisting psychiatric diagnoses, except for personality disorder diagnoses. The analyses were also adjusted for age at the first personality disorder diagnosis, age at first delivery and year of birth.

### Statistical analyses

Statistical analyses were conducted using SAS 9.2 for Windows (SAS Institute Inc, Cary, NC, USA). Crude and multivariate analyses were performed using Cox regression models of time to death during follow-up. We assessed person-years at risk by totalling the years that the individuals were alive during the follow-up period. The entry date was defined as the date of first personality disorder diagnosis, and the exit date as the date of death or the end of follow-up (31 December 2016). Two regression models were examined: model I, which adjusted for year of birth and education level, and model II, which adjusted for age at diagnosis, psychiatric and somatic comorbidity and severity of the personality disorder in addition to the year of birth and education level.

## Results

The 27 412 women included in the study received their first diagnosis of personality disorder at a mean age of 25.8 years (s.d. = 6.1 years) (Table 1). In total, 59% (or 16 096) of the women were nulliparous and 11 318 parous. Slightly more than one-third of the cohort (i.e. 37%) had <10 years of education, 39% had 10–12 years and 24% had a college or university education.

**Table 1:** Cohort characteristics of the 27 412 women born between and including the years 1971 and 1995 who were diagnosed with a personality disorder in 1990–2015

	All	Childbearing status		
		Nulliparous	Childbearing after personality disorder diagnosis	Childbearing before personality disorder diagnosis
All, *n* (row %)	27 412 (100)	16 094 (59)	4556 (17)	6762 (25)
Age at first diagnosis, mean (s.d.)	25.8 (6.1)	24.5 (5.4)	22.9 (4.3)	30.9 (5.7)
Age at first delivery, ^a^mean (s.d.).	24.9 (5.1)	N/A	27.5 (4.9)	23.1 (4.4)
Age at death, mean (s.d.)	29.9 (6.4)	28.4 (6.0)^b^	31.7 (5.3)^b^	34.1 (6.0)^b^
Year of birth, *n* (%)^c^				
1971–1975	5357 (20)	1996 (12)	850 (19)	2511 (37)
1976–1980	5559 (20)	2451 (15)	1151 (25)	1957 (29)
1981–1985	6315 (23)	3518 (22)	1398 (31)	1399 (21)
1986–1990	6475 (24)	4776 (30)	977 (21)	722 (11)
1991–1995	3706 (14)	3353 (21)	180 (4)	173 (3)
Education, *n* (%)^c^				
Compulsory school (≤9 years)	10 244 (37)	6159 (38)	1467 (32)	2618 (39)
High school (10–12 years)	10 695 (39)	5951 (37)	1827 (40)	2917 (43)
College or university (≥13 years)	6448 (24)	3959 (25)	1262 (28)	1227 (18)
Missing	25 (0)	25 (0)	0 (0)	0 (0)
Severity, *n* (%)^c^				
Out-patient only	15 411 (56)	9060 (56)	1982 (44)	4369 (65)
In-patient	12 001 (44)	7034 (44)	2574 (56)	2393 (35)
Psychiatric comorbidity^c^, *n* (%)	22 181 (81)	13 042 (81)	3720 (82)	5419 (80)
Substance abuse disorders^d^	6138 (22)	3658 (23)	1107 (24)	1373 (20)
Schizophrenia/non-affective psychotic disorders	1987 (7)	1402 (9)	269 (6)	316 (5)
Depressive disorders	11 805 (43)	6760 (42)	2061 (45)	2984 (44)
Anxiety disorders	14 426 (53)	8373 (52)	2541 (56)	3512 (52)
Behavioural disorders	5624 (21)	3288 (20)	882 (19)	1454 (22)
Other psychiatric disorders	5848 (21)	3855 (20)	1026 (23)	967 (14)
Somatic comorbidity, *n* (%)	10 797 (39)	5697 (35)	2483 (54)	2617 (39)

N/A Not applicable.

a. In parous women.

b. *P* < 0.05 between the three groups: ANOVA and Duncan's *post hoc* tests.

c. Column percent of all patients.

d. As several types of comorbidities may occur the figures add to more than 100%.

In all, 692 women died during the study period. Of these women, 126 (18%) died because of natural causes and 566 (82%) because of unnatural causes. The dominating unnatural cause of death was suicide (*n* = 472, 68%).

Nulliparous women had a higher risk of mortality than those with a childbearing history for both natural and unnatural deaths (Table 2). In model I, in which year of birth and education were adjusted, the hazard ratio (HR) for death in nulliparous women versus parous women was 1.71 (95% CI 1.44–2.03). In model II, in which age at diagnosis, psychiatric and somatic comorbidity and severity were adjusted in addition to the year of birth and education, the HR remained about the same 1.78 (95% CI 1.50–2.12). The risk of death was most pronounced for suicide, with HRs close to 2.

**Table 2:** Hazard ratios with 95% CIs for all-cause mortality, natural and unnatural deaths and suicides as regards parity in women with a personality disorder

	Hazard ratio (95% CI)	
	Delivered	Nulliparous
All-cause mortality		
Rate per 100 000 person-years	279.1 (242.9–320.7)	402.6 (368.6–439.7)
Model I^a^	1 (REF)	1.71 (1.44–2.03)
Model II^b^	1 (REF)	1.78 (1.50–2.12)
Natural		
Rate per 100 000 person-years	56.1 (41.2–76.5)	70.2 (56.9–86.8)
Model I^a^	1 (REF)	1.73 (1.17–2.55)
Model II^b^	1 (REF)	1.94 (1.31–2.88)
Unnatural		
Rate per 100 000 person-years	223.0 (190.9–260.5)	332.4 (301.6–366.3)
Model I^a^	1 (REF)	1.70 (1.41–2.06)
Model II^b^	1 (REF)	1.75 (1.44–2.12)
Suicide		
Rate per 100 000 person-years	161.3 (134.4–193.6)	291.5 (262.8–323.4)
Model I^a^	1 (REF)	2.09 (1.68–2.60)
Model II^b^	1 (REF)	2.13 (1.71–2.66)

REF, reference.

a. Adjusted for year of birth and education.

b. Adjusted for year of birth, education, age at diagnosis, psychiatric and somatic comorbidity and severity.

Considerable variability in age at first personality disorder diagnosis and age at first delivery was observed, factors that we assumed would represent individual differences in life trajectories. There were also substantial time differences between the first delivery and the diagnosis of personality disorder in parous women. Thus, the absolute difference between age at diagnosis and age at first delivery, i.e. without considering which occurred first, was 5.6 years (s.d. = 4.9 years, data not shown). Moreover, age at first delivery, but not age at diagnosis, had an independent effect on mortality in the parous women, and without any significant interaction between these two factors (Table 3).

**Table 3:** Cox regression with both age-related variables and an interaction variable (age at delivery × age at first personality disorder diagnosis) in parous women adjusted for year of birth and education

	All-cause mortality	Natural	Unnatural	Suicide
Age at delivery	0.77 (0.66–0.89)	0.69 (0.49–0.96)	0.79 (0.67–0.93)	0.77 (0.64–0.93)
Age at first personality disorder diagnosis	0.92 (0.81–1.04)	0.81 (0.62–1.06)	0.95 (0.83–1.09)	0.93 (0.79–1.08)
Interaction variable	1.01 (1.00–1.01)	1.01 (1.00–1.02)	1.01 (1.00–1.01)	1.01 (1.00–1.01)

A majority (60%, mean age 24.9 years, s.d. = 5.1) had given birth before being diagnosed with personality disorder (6762/11 318). For clinical reasons, we subsequently divided the cohort into those who gave birth before versus after the personality disorder diagnosis and assessed whether those who gave birth before had a higher mortality risk (Table 4). Nulliparity was associated with a twofold increased risk for death compared with giving birth after the first personality disorder diagnosis (adjusted HR = 2.29, 95% CI 1.72–3.04) and giving birth before a first personality disorder diagnosis was associated with a 1.5-fold increased risk. The elevated HR of suicide was threefold in the nulliparous women compared with those giving birth after their first personality disorder diagnosis (adjusted HR = 2.90, 95% CI 1.97–4.26). However, the higher death risk in those who gave birth before diagnosis than in those who did not was only observed for unnatural deaths and suicide but not for natural deaths.

**Table 4:** Hazard ratios with 95% CIs for all-cause mortality, natural and unnatural death and suicide as regards parity in women with a personality disorder by comparison group

	Hazard ratio (95% CI)		
	Delivered after first personality disorder diagnosis	Nulliparous	Delivered before first diagnosis
All-cause mortality			
Rate per 100 000 person-years	185.9 (143.1–241.6)	402.6 (368.6–439.7)	347.2 (294.8–409.1)
Model I^a^	1 (REF)	2.35 (1.77–3.11)	1.63 (1.19–2.24)
Model II^b^	1 (REF)	2.29 (1.72–3.04)	1.48 (1.06–2.07)
Natural			
Rate per 100 000 person-years	46.5 (27.5–78.5)	70.2 (56.9–86.8)	63.1 (43.0–92.7)
Model I^a^	1 (REF)	1.90 (1.06–3.41)	1.16 (0.59–2.27)
Model II^b^	1 (REF)	1.76 (0.97–3.21)	0.86 (0.42–1.77)
Unnatural			
Rate per 100 000 person-years	139.5 (103.1–188.7)	332.4 (301.6–366.3)	284.1 (237.0–340.5)
Model I^a^	1 (REF)	2.48 (1.81–3.42)	1.78 (1.25–2.55)
Model II^b^	1 (REF)	2.45 (1.77–3.39)	1.70 (1.17–2.49)
Suicide			
Rate per 100 000 person-years	96.3 (66.9–138.6)	291.5 (262.8–323.4)	208.8 (169.1–258.0)
Model I^a^	1 (REF)	3.03 (2.07–4.43)	1.91 (1.25–2.92)
Model II^b^	1 (REF)	2.90 (1.97–4.26)	1.70 (1.08–2.66)

REF, reference.

a. Adjusted for year of birth and education.

b. Adjusted for year of birth, education, age at diagnosis, psychiatric and somatic comorbidity and severity.

## Discussion

### Main findings

Although the challenges encountered by women with personality disorder have been extensively documented for pregnant women and those giving birth,^26,35^ this study focused on a less studied phenomenon, namely the effect of childbearing on mortality in women with personality disorder. The main finding was that nulliparous women with personality disorder have a higher mortality risk than parous women with personality disorder, regardless of whether childbearing had occurred before or after the personality disorder diagnosis. This finding occurred for both natural and unnatural causes of death (especially suicide). The lower mortality in childbearing women, however, was more pronounced in those who gave birth after the personality disorder diagnosis than in those who gave birth before the diagnosis. All-cause mortality risk was over two times higher in women who did not give birth at all and 1.5 times higher in women who gave birth before the personality disorder diagnosis than those who gave birth post-diagnosis.

Interpretation of our results

Individuals with a diagnosis of personality disorder have an excess risk of all-cause and cause-specific mortality, with an increased risk for unnatural causes and suicide being most pronounced.^3,4,36^ This feature can be viewed as the product of all the difficulties encountered in everyday life, including problems with interpersonal relationships and social interactions, with affect regulation or cognition and with comorbidity. The lower death risk in childbearing women can either reflect a protective effect of the childbearing process as such or that the women giving birth may have better health before pregnancy. Still, there was a lower death risk in childbearing women even after adjustment for comorbidity and in-patient care, both of which represent more serious conditions.^3,4^

Childbearing is related to both lower overall mortality in the general population^11,12^ and a lower risk for suicide.^14,15^ For example, one study from Australia showed that all-cause mortality in women decreased with increasing parity.^11^ Similar findings were reported in a Japanese study,^12^ in which parous women had an HR of 0.67 (95% CI 0.46–0.97) for all-cause mortality. A fairly recent meta-analysis showed that those with no live birth had a significantly elevated risk of all-cause mortality compared with participants with one or more live births.^13^

On the other hand, early teenage pregnancy is a social risk factor with increased mortality risk.^23^ Moreover, women with personality disorder are at a higher risk of developing psychiatric symptoms during the perinatal period^19,22^ and personality disorder has been linked to a higher prevalence of perinatal complications.^26^ Taken together, childbearing and motherhood in patients with a diagnosed personality disorder may be both protective and a risk factor, depending on the individual and the context.

A key feature in the diagnosis of borderline personality disorder,^37^ which constitutes a substantial part of all patients diagnosed with personality disorder, is a chronic feeling of emptiness. In this group, a feeling of meaningfulness in life has been found to play a buffering role against hopelessness and to be a protective and preventive factor for suicide.^38^ From an overall evolutionary perspective, childbearing contains substantial meaningful components, which may contribute to a better psychiatric well-being.

The finding that the beneficial effect was less pronounced, although still present, in the group giving birth before the personality disorder diagnosis, and at an earlier age than those giving birth after their diagnosis, suggests that this group is burdened by the risks associated with early pregnancy. Low maternal age is linked to a higher rate of unwanted pregnancies and mothers worse equipped to meet the challenges of childbearing. Some evidence suggests that teenage mothers confront an increased risk of premature death later in life compared with adult mothers and that postpartum suicide attempts are associated with younger age.^24,39^ At the same time, those giving birth before the clinical diagnosis of personality disorder obtained their diagnosis at an older age than those who gave birth after diagnosis. Based on our results, higher age at diagnosis seems to be less associated with the risk of death. The present data further suggest that age at a first birth-giving is a dominant factor for the less beneficial effect in the group giving birth before the diagnosis of personality disorder.

Mediating factors that need to be considered include various aspects of support provided by professional healthcare providers. Women diagnosed with a personality disorder will thus be recognised within the maternity care services, which enables substantial support during pregnancy and early infancy. In the same way one can assume that women with personality disorders will also receive more focused support from the psychiatric services.

### Strengths and limitations

A strength of this study is the use of population-based national registers with high coverage and validity, including for personality disorders.^27,28^ In addition, the large cohort allowed for detailed analyses of different subgroups of childbearing and causes of death. Nevertheless, there are limitations. A first limitation concerns the lack of total agreement between ICD-9 and ICD-10 regarding the criteria for different personality disorders. This lack of agreement, however, is of less importance for mortality.^3^

A second limitation is that the registered diagnoses are given at the discretion of the treating physician, which is why its scientific validity for individual patients can be questioned. Furthermore, based on epidemiological data, only a small fraction of individuals who fulfil symptoms of personality disorder obtain a diagnosis in clinical care. Thus, the results cannot be generalised beyond the group of individuals who are formally diagnosed. Finally, with register-based data, there are various factors and confounders that we were not able to consider in the analyses, including subtle differences in the clinical presentation or the course of symptoms before the formal first diagnosis.

### Implications

The finding that childbearing is related both to less natural and unnatural death in women with a personality disorder needs to be taken into account when evaluating patients, specifically when assessing risk for suicide. Another implication of the observations made in this study is that they help to understand the issue and can be used as a basis for further research on the specific factors of motherhood that carry the lower mortality observed.

In conclusion, this study found that nulliparous women with personality disorder have a higher mortality risk than parous women with personality disorder, regardless of whether childbearing had occurred before or after the personality disorder diagnosis. This effect of childbearing, however, was more pronounced in women who gave birth after the personality disorder diagnosis than in those who gave birth before the diagnosis. These findings suggest that childbearing history should be an integral part of the clinical evaluation of women with personality disorder.

## Data Availability

The data that support the findings of this study are not publicly available. According to the Swedish Ethical Review Act, the Personal Data Act and the Administrative Procedure Act, data can only be made available after a legal review for researchers who meet the criteria for access to this type of sensitive and confidential data.
